# Supplementary material for: 100 Hz ROCS microscopy correlated with fluorescence reveals cellular dynamics on different spatiotemporal scales
Source: Nat Commun. 2022 Apr 1;13:1758. doi: 10.1038/s41467-022-29091-0 (PMC8975811; doi:10.1038/s41467-022-29091-0)
Supplement: Supplementary file 1 — Supplementary Information [file 41467_2022_29091_MOESM1_ESM.pdf]

Supplementary Information to

100 Hz ROCS microscopy correlated with fluorescence reveals cellular dynamics on different spatiotemporal scales

Felix Jünger[1], Dominic Ruh[1], Dominik Strobel[1], Rebecca Michiels[1], Dominik Huber[1], Annette Brandel[2,3], Josef Madl[3,4,5], Alina Gavrilov[7], Michael Mihlan[7], Caterina Cora Daller[4], Eva A. Rog-Zielinska[4,5], Winfried Römer[2,3,6], Tim Lämmermann[7], Alexander Rohrbach[1,2,3,5]*

[1]*Laboratory for Bio- and Nano-Photonics, Department of Microsystems Engineering, University of Freiburg*
[2]*Faculty of Biology, University of Freiburg, Schänzlestraße 1, 79104 Freiburg, Germany*
[3]*Signalling Research Centres BIOSS and CIBSS, University of Freiburg, Schänzlestraße 18, 79104 Freiburg, Germany*
[4]*Institute for Experimental Cardiovascular Medicine, University Heart Center Freiburg · Bad Krozingen, and Faculty of Medicine, University of Freiburg, Elsässer Straße 2q, 79110 Freiburg, Germany*
[5] *German Collaborative Research Centre SFB1425 University of Freiburg, Freiburg, Germany*
[6] *Freiburg Institute for Advanced Studies (FRIAS), University of Freiburg, 79104 Freiburg, Germany*
[7] *Max Planck Institute of Immunobiology and Epigenetics, Stübeweg 51, 79108 Freiburg, Germany*
*Corresponding author: rohrbach@imtek.de

Supplementary Note 1: Optical setup of ROCS microscope

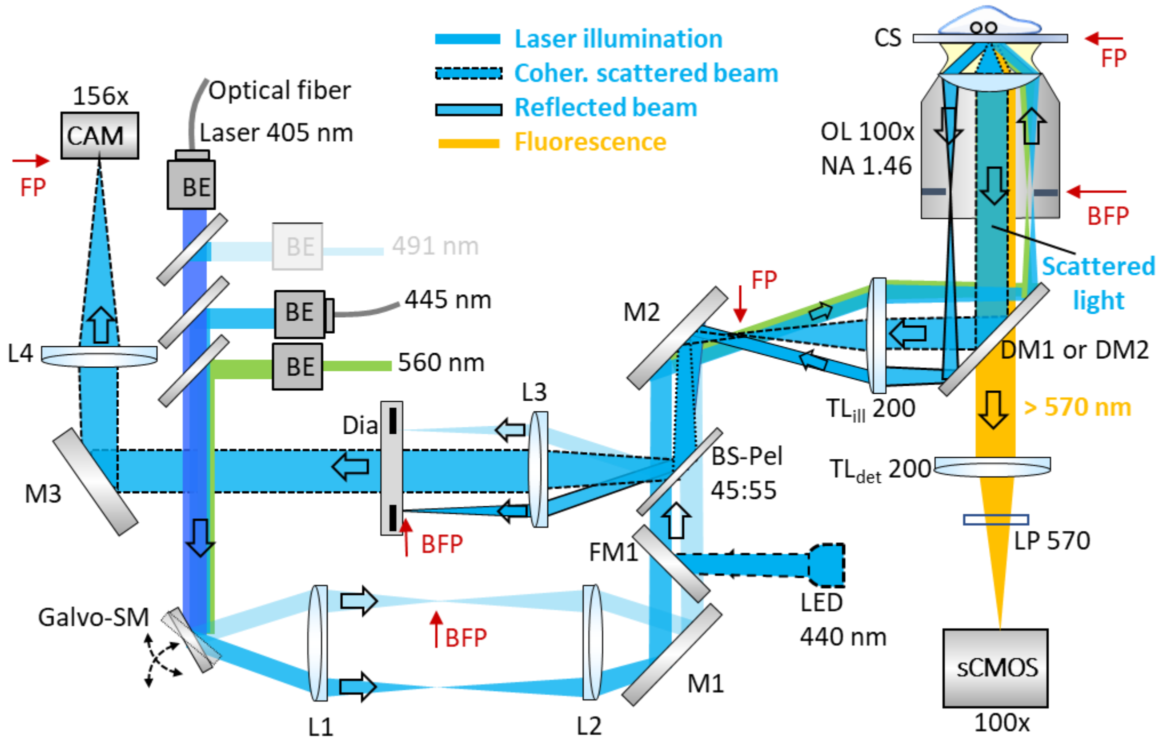

Figure S 1 | Sketch of the ROCS setup used in this study (not to scale). Diode laser outputs (405 nm and 445 nm fiber-coupled, 491 nm and 560 nm free space) are expanded by a 3x beam expander (BE) and deflected in azimuthal angles by galvanometric scan mirrors (SM) and imaged by lenses (L1, L2, TL-ill, OL) into the image plane of a 100x objective lens (OL). The rotating, oblique, collimated laser light is scattered at the sample in the focal plane (FP) and collected by the OL. The pellicle beam splitter (BS-pel) reflects 45% of the light collected by the OL and thus separates the detection and illumination beam path. A variable darkfield diaphragm (dia) in the pupil plane (BFP) can be used to block the unscattered illumination light to enable darkfield imaging with the CMOS-camera (cam). All lasers can be used to excited fluorescence light, which is imaged onto a sCMOS camera. LED illumination can be added using a flip mirror (FM1).

A ROCS imaging system does not require many components and can be implemented into an existing microscope or built from scratch as a standalone microscope. The ROCS microscope in this study is based on an inverted microscope (Leica DM-IRBE). This setup is designed to accommodate both ROCS and total internal reflection fluorescence (TIRF) microscopy using the same microscope. Both the illumination beam paths for ROCS and TIRF are coupled into the microscope's rear port. ROCS illumination is possible with a 405nm diode laser (Oxxius LBX-405, France), a 445nm diode laser, (Cobolt SE, Sweden) and a 491nm diode laser (Cobolt SE, Sweden). Fluorescence can be excited with 491nm and 561nm diode lasers (Cobolt SE, Sweden).

ROCS illumination beam path

Light blue beams indicate the course of the ROCS laser beam. Incident beam and the light scattered at the sample have the same wavelength λ_0, as displayed in Figure S 1 in dark blue and purple respectively for better visibility. The laser output is coupled into a single mode fiber (Thorlabs, 405 PM-S405-XP // Coupling-In F671FC-405 // Coupling-Out AC080-010-A-ML) to clean up the non-Gaussian beam profile of the laser diode output and generate a Gaussian beam profile of ellipticity 0.99. The beam is then threefold expanded by a 4f system (BE, two achromatic lenses of focal length 20 mm and 60 mm, Edmund Optics VIS0°) to a diameter of 2.7 mm, which fills the aperture of the subsequent scan mirrors (Sunny Technologies, S8210M). The scan mirrors rotate the laser beam along a circular trajectory to achieve oblique illumination at various azimuthal directions. This scan mirrors can deflect the beam in both lateral axes x-y by a maximum angle of ± 12.5° at frequency up to 300 Hz. The deflected beam is further expanded to 5 mm using another 4f system consisting of achromatic lens L1 with f1 = 75 mm and achromatic lens L2 with f2 = 140 mm (both Edmund Optics VIS0°) to optimize the illumination field of view. A 45:55 (R:T) pellicle beam splitter (BS-Pel; BP245B1, Thorlabs, Newton, NJ, USA) transmits 55% of the incident illumination light into the microscope.

Fluorescence illumination beam path

The (TIR)-ROCS setup is combined with a (TIR)-Fluorescence microscope with 491nm and 561nm diode lasers (Cobolt SE, Sweden) and a 440nm LED (3W, emitter surface = 1mm²). Basic beam alignment is achieved by mounting the dichroic mirror on a kinematic mount and combine it with another kinematic mounted mirror M2 placed after the pellicle BS-Pel. Using this configuration, it is possible to guide the beam parallel to the optical axis of the illumination tube lens as well as adjust any offset to the rear port of the Leica microscope.

All lenses in the ROCS beam path are VIS0° lenses (Edmund Optics) with a reflectivity of only 0.2% for the used wavelengths. This strongly reduces unwanted reflections and image background. Illumination tube lens TL_{ill} (Edmund optics) with f_{TL} = 200 mm and objective lens OL (HCX PL Apo, 100x, NA 1.46, oil immersion, Leica Microsystems, Wetzlar Germany) achieve a magnification of M = 100. The mainly used dichroic mirror (DM1) (F68-406, AHF Analysentechnik AG, Tübingen, Germany) inside the microscope body reflects the ROCS and transmits fluorescence light (for GFP and mCherry) to an sCMOS camera (ORCA-Flash 4.0 V2, Hamamatsu Photonics, Japan). As a result, it is possible to acquire both ROCS and fluorescence image simultaneously. The OL is optimized for TIR-F and is capable to collect light under a maximum angle for n_i = 1.52. The OL is mounted on a z-piezo (MIPOS 100 UD SG, Piezosystem Jena) that can be moved ±50 µm along the optical axis with a precision of 2 nm to enable fine alignment. The illumination beam is focused by TL_{ill} at the TIR ring of the BFP of OL, the plane wave emanating from the objective lens undergoes TIR at the interface between glass cover slip and sample immersed in water. The resulting evanescent wave at the interface decays exponentially along axial direction and therefore excites only a thin section of the sample in TIR mode.
Camera pixel size Orca Flash: 1px = 6.45µm, at magnification M = 100; i.e. one pixel in the image is 64.5nm

Detection beam path

The coherently scattered light is collected by the same objective lens and tube lens used for illumination. The pellicle beam splitter BS-Pel (R=45%) separates the detection beam path from the

illumination beam path. A 4f system consisting of two achromatic lenses L3 with *f3* = 160 mm and L4 with *f4* = 250 mm images the beam onto a fast CMOS (Sony IMX174 sensor) camera (PointGrey GS3-U3-23S6M-C, Richmond BC, Canada). This configuration allows access to a pupil plane (PP-DF) conjugated to the BFP without interfering with the illumination beam path. The 12 bit-camera exhibits the best compromise of good low-light performance, fast frame rates (163 Hz full-chip) and decent quantum efficiency (> 50%).

Camera pixel size PointGrey: 1px = 5.86µm, at magnification M = 100*250/160 = 156; i.e. one pixel in the image is 37.5nm

Supplementary Note 2: Brightfield vs. Darkfield ROCS imaging

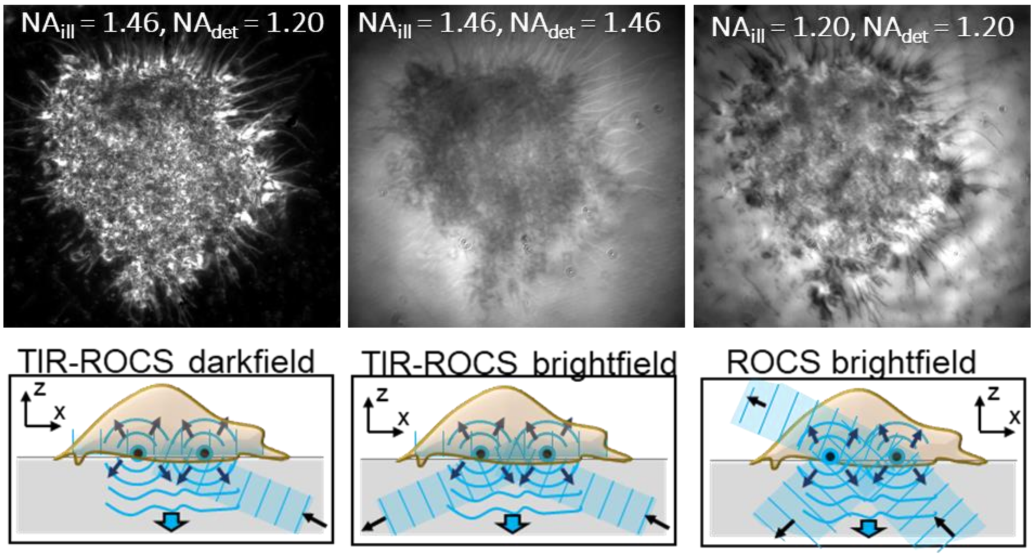

Figure S 2 | Influence of brightfield and darkfield mode on the ROCS image. Upper row, from left to right: TIR-ROCS image of the same J774 macrophage. Left: darkfield image with NA_{det} = 1.2. Middle: brightfield image with NA_{det} = 1.46, Right: brightfield image with NA_{det} = 1.2. Sketches in the lower row illustrate the difference in image formation of both modes.

Figure S 2 illustrates that the choice of brightfield (BF) and darkfield (DF) mode has a tremendous influence on the resulting (TIR)-ROCS image. Both images were obtained from the same sample (J774 macrophage, adherent on a standard microscope coverslip) and taken with the same illumination wavelength (405nm) at a laser power of < 1mW. The switch between BF and DF is done by closing the variable aperture in the detection beam path (see Supplementary Note 1 for details). In DF mode, the aperture blocks the directly reflected light, thus reducing the effective detection numerical aperture NA_{det} to approximately 1.2. The resulting image allows a high-contrast view of the sample with excellent SNR. Especially all the adherent filopodia that surround the cell body can be displayed with optimal contrast in DF mode, see upper left image panel. The brightfield image without a DF aperture allows to image with the full NA_{det} = 1.46, i.e. with maximal lateral image resolution. In BF mode, additional interferences of the (unscattered) reflected light and the scattered light contribute to the final image (see concept sketches in the lower row). Switching the deflection angle of the galvanometric mirrors (see Supplementary Note 1 for details) allows to alter the illumination NA_{ill}. The right panel in the upper row shows a brightfield image with reduced NA_{ill} and NA_{det}. The transition of bright and dark image intensities from interferences, occur mainly at the base of the filopodia / lamellipodia, where the sample thickness changes.

We have shown three different example images to illustrate the possibilities that illumination with different NAs and imaging modes. We envision that a combination of different imaging modalities

during ROCS acquisition will provide access to additional sample information, such as sample height, thickness, distance to coverslip, curvature, just to name a few.

Supplementary Note 3: ROCS Imaging of 150nm beads

Using polysterene beads with a mean diameter of 150nm (Thermo Scientific™ Fluoro-Max blue) we could demonstrate a 150nm spatial resolution in darkfield TIR-ROCS microscopy. Using a reduced detection NA_{det} = 1.25 and a larger illumination NA_{ill} = 1.45 at a wavelength λ = 405nm, the formula $\Delta x = \lambda / (NA_{det} + NA_{ill})$ = 150nm provides a good resolution estimate. Four different linescans show the separability of adjacent bead intensities in Figure S2.

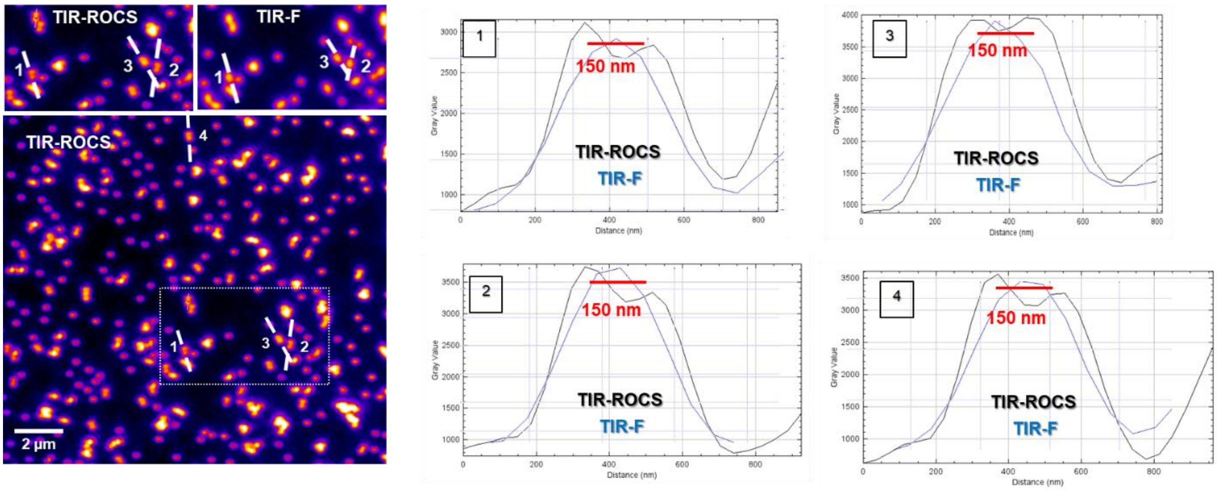

Figure S 3 | TIR-ROCS and TIR-F images of 150nm beads. Left: Polysterene beads imaged at λ = 405nm in darkfield ROCS mode at NA_{det} = 1.2 and NA_{ill} = 1.4 are shown in pseudo colors. A region of interest is magnified and compared to a TIR-F image (λ = 505nm, NA = 1.46). Four exemplary linescans, numbered by 1..4, are displayed on the right side indicating a distance of about 150nm between ROCS-peaks. Beads cannot be resolved with TIR-F (blue lines).

Supplementary Note 4: Spatially and temporally coherent illumination

As shown in the previous supplementary section, resolution and contrast are strongly controlled by the detection numerical aperture NA_{det} and the illumination angle described by NA_{ill}. However, in coherence or interference microscopy the degree of spatial and temporal coherence plays a significant role as well as illustrated by the following two figures. The subsequent addition of intensity images from different azimuthal angles 0…2π is always incoherent

$$p_{ROCS}(x,y) = \int_0^{2\pi} \left|E_{cam}(x,y,\phi)\right|^2 d\phi\,,$$

But, an image from a single illumination direction ϕ_0 depends on the (temporal and spatial) coherence, i.e. the variation of the polar angle $\varepsilon = \Delta\theta$, or the variation of the wavelength $\varepsilon = d\lambda$:

$$p_{\phi_0}(x,y,\varepsilon) = \int \left|E_{cam}(x,y,\phi_0,\varepsilon)\right|^2 d\varepsilon = \int \left|E_i\left[\left(e^{-i\mathbf{k}_i(\phi_0,\varepsilon)\mathbf{r}}\right)\cdot f(x,y)\right] * PSF_A(x,y)\right|^2 d\varepsilon$$

Here $f(x,y)$ describes the object, $PSF_A(x,y)$ the electric field pointspread function and $E_i \cdot e^{-i\mathbf{k}_i(\phi_0,\varepsilon)\mathbf{r}}$ the electric field coming from a direction, defined by the incident wave vector $\mathbf{k}_i$.
Figure S 3 illustrates the coherent diffraction of plane waves at two particles under different angles $\varepsilon = \Delta\theta$, resulting in fringe patterns from Fraunhofer diffraction. These patterns are slightly shifted with respect to $\Delta\theta$, such that their (incoherent) summation of (intensity) diffraction patterns results in a

pattern with reduced fringe contrast (black pattern on the right, relative to a green pattern as a reference). A light source with a reduced spatial coherence such as from LEDs reduces the contrast of the fringes and of the corresponding Fourier transforms in the image plane, respectively.

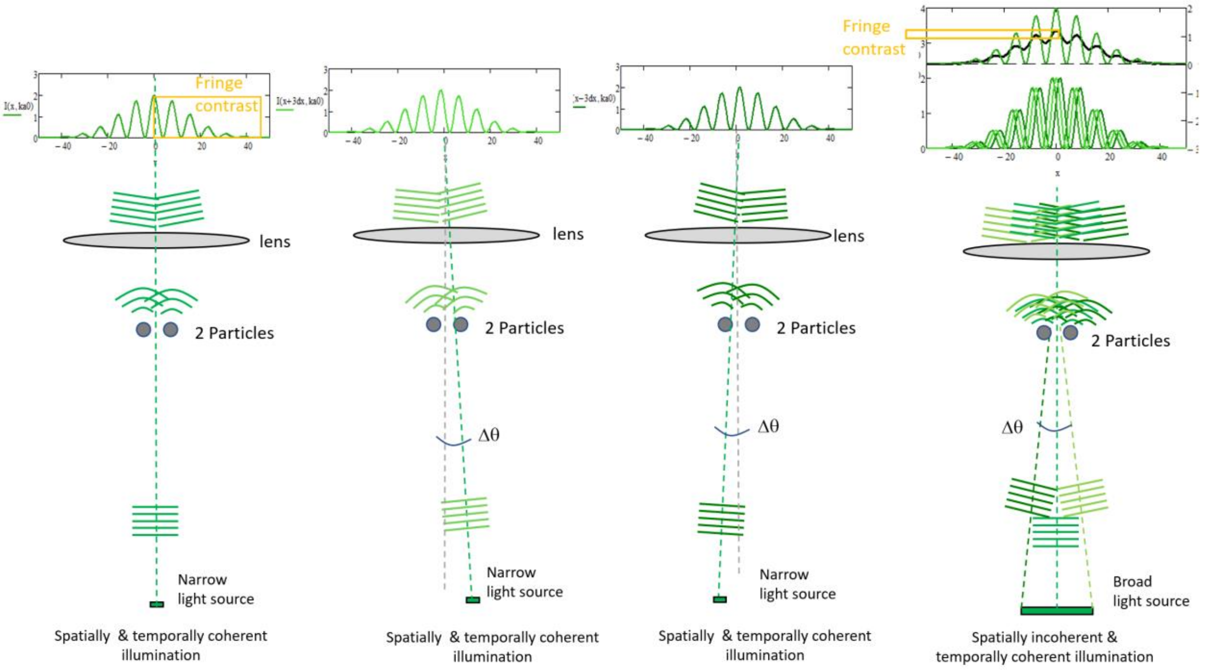

Figure S 4 | Effect of spatial coherence on contrast. Spatially and temporally coherent illumination (from a laser) results in diffraction patterns with high fringe contrast. Different angles of illumination result in different shifts of the intensity patterns. Summation of the fringe patterns results in strongly reduced fringe contrast, as a result of spatially incoherent illumination from different angles, although the green laser wavelength is not changed.

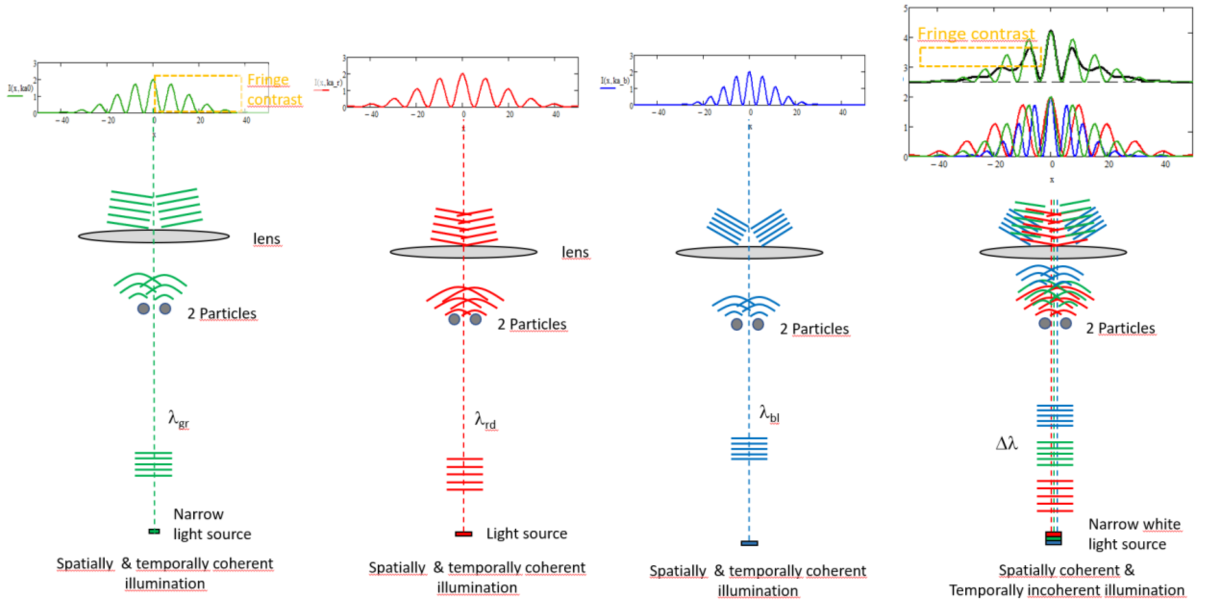

Figure S 5 | Effect of temporal coherence on contrast. Spatially and temporally coherent illumination (from a laser) results in diffraction patterns with high fringe contrast. Different illumination wavelengths (550nm, 700nm and 450nm) result in different intensity patterns regarding fringe frequency and width. Summation of the fringe patterns results in strongly reduced fringe contrast, as a result of temporarily incoherent illumination from wavelengths, although the illumination direction is not changed.

Figure S 4 illustrates the coherent diffraction of plane waves at two particles under different wavelengths $\varepsilon = d\lambda$, resulting in fringe patterns from Fraunhofer diffraction. These patterns change in width and frequency with respect to $\Delta\lambda$, such that their (incoherent) summation of (intensity)

diffraction patterns results in a pattern with reduced fringe contrast (black pattern on the right, relative to a green pattern as a reference). A light source with a reduced temporal coherence such as from LEDs reduces the contrast of the fringes and of the corresponding Fourier transforms in the image plane, respectively.

Supplementary Note 5: Influence of the spatial coherence on ROCS images

Figure S 6 compares images of the same J774 macrophage taken with different modes of illumination, resulting in different degrees of spatial and temporal coherence. Panel a shows a single-shot image of a cell required with a 440nm LED in epi-illumination mode at polar angle θ = 0°, normal incidence).

Using a 445nm laser illumination at the same normal incidence θ = 0° (without laser rotation), the cell is remains nearly invisible in panel b because of a too strong background from unscattered light. The cell but becomes slightly visible in panel c after post-processive background (BG) subtraction. In panel d we reduced the spatial coherence of the laser light by a rotating diffuser wheel in the ROCS beam path close to the fiber output (see Supplementary Note 1 for details). While the image taken with the LED (panel a) is characterized by both limited spatial and temporal coherence, the temporal coherence in panel b is maximal, resulting in speckle structures despite the reduced spatial coherence.

When the illumination laser rotates at 100 Hz at a fixed polar angle θ, images of different azimuthal angles are added up incoherently during the 10 ms integration time of the camera. This additional variation of the spatial coherence (azimuthally incoherent) allows to decipher the cell against a still prominent background of unscattered laser light as shown in panel e (laser rotating at θ = 50°). Subtracting the Gaussian intensity background generates an image which shows significantly more cellular details like filopodia, but still does not reach the image quality of the LED image in panel a.

For highly oblique incidence at θ = 70° (evanescent TIR illumination), the darkfield condition is fulfilled where $NA_{ill} > NA_{det} = 1.2$. At such high illumination angles many cellular structures become visible with high contrast and resolution. Additional background subtraction discloses even more cellular details at a spatial resolution of about 0.15µm.

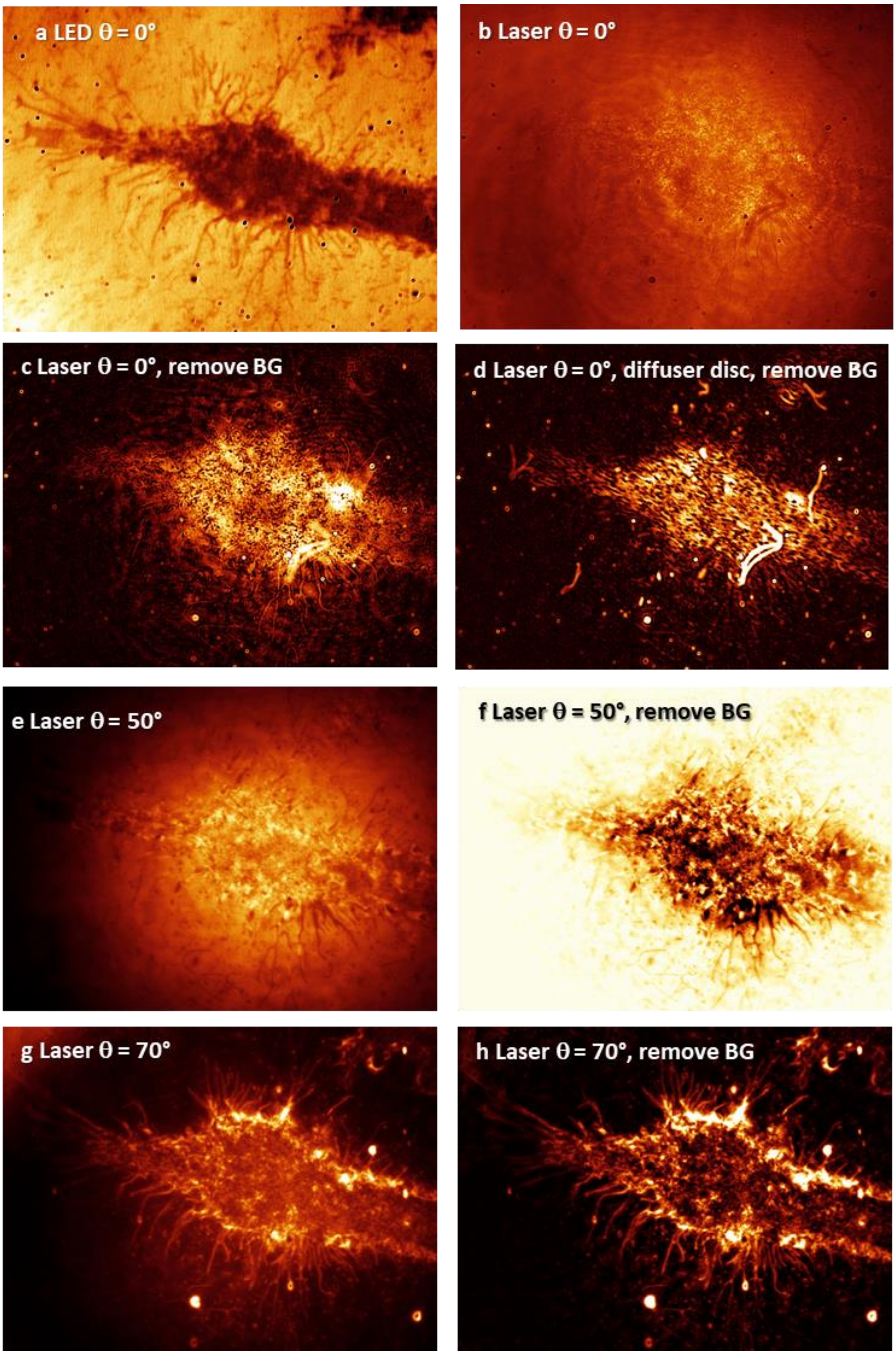

Figure S 6 | Changing the illumination mode. Influence of polar angle and spatial coherence on the resulting ROCS images at NA_{det} = 1.2 of. Sample: J774 macrophage. Illumination was performed with an incoherent LED (**a**) and with a coherent 405nm laser source (**b-h**)and with three different illumination polar angles θ. **d,f,h** Background subtraction reveals more cellular details.

Supplementary Note 6: background subtraction and activity maps

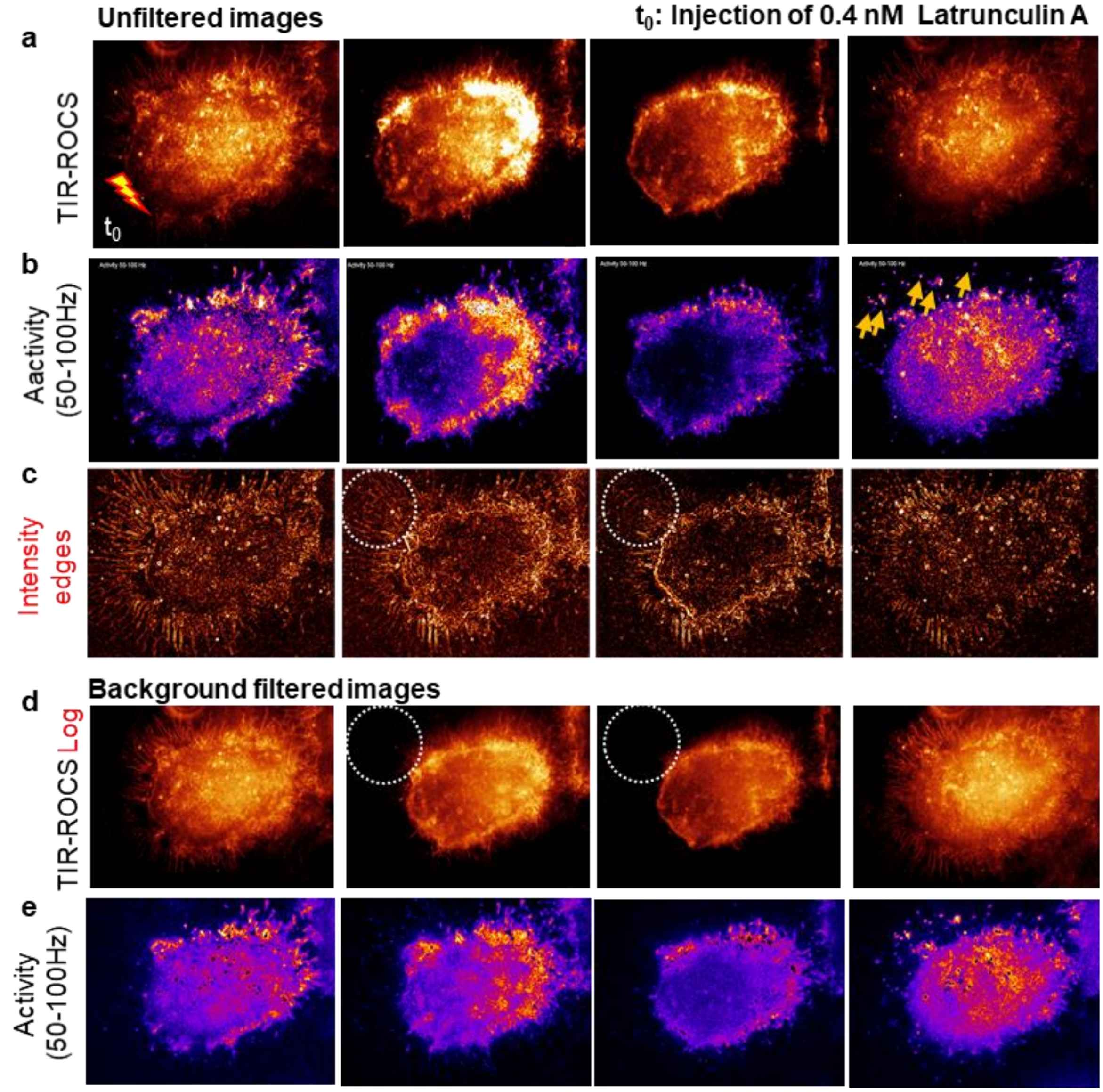

Figure S 7 | Effect of background subtraction on ROCS image details and activities - Supporting images to Figure 2. The images show the fast whole-cell reaction of a J774 macrophage upon injection of low concentration Latrunculin A. Columns show the same observation time points as in Figure 2 (from left to right). **a** Unfiltered raw images show strong scattering signals at the cell periphery. **b** Enhanced activity is visible at bright and less bright images in the cell cortex. **c** Enhanced edges of ROCS intensity images emphasize the structure of filopodia especially in round ROIs (Find edges in ImageJ). **d** ROCS images of panel **a** are background subtracted (low pass filtering) and displayed in logarithmic scales, without revealing filopodia or membrane tubes of filopodia. Images appear without increased intensity areas. **e** Activity maps from background filtered images show the same regions of enhanced activity, indicating that the ROCS signal strength has only a minor influence on the activity.

The data in Figure S 7 supplements the data shown in Figure 2 in the main text (fast whole-cell reaction of a J774 macrophage upon treatment with a small amount of Latrunculin A). The original dataset is the same as shown in the main text. The columns show the same observation time points as in Figure 2 (from left to right). In addition to Figure 2, the logarithmic TIR-ROCS images are shown (panel d). In order to rule out any artificial effects of an uneven image background on the activity calculation, the images were filtered with the ImageJ standard filter routine in order to remove any remaining background. Activity maps are calculated with the same parameters again and are shown in panel e. We note that there is only a small difference between activity of the raw data (panel b) and that of

background-filtered data (panel d). We conclude that the activity is not an artefact that possibly comes from an uneven image backgrounds or strong ROCS scattering signals. Panel c shows the edges of cellular structures, especially filopodia (Find edges in ImageJ). This representation shows low and high rocs signals in a comparable way hence making even very thin filopodia or membrane tubes from filopodia with retracted backbone visible.

Activity background subtraction

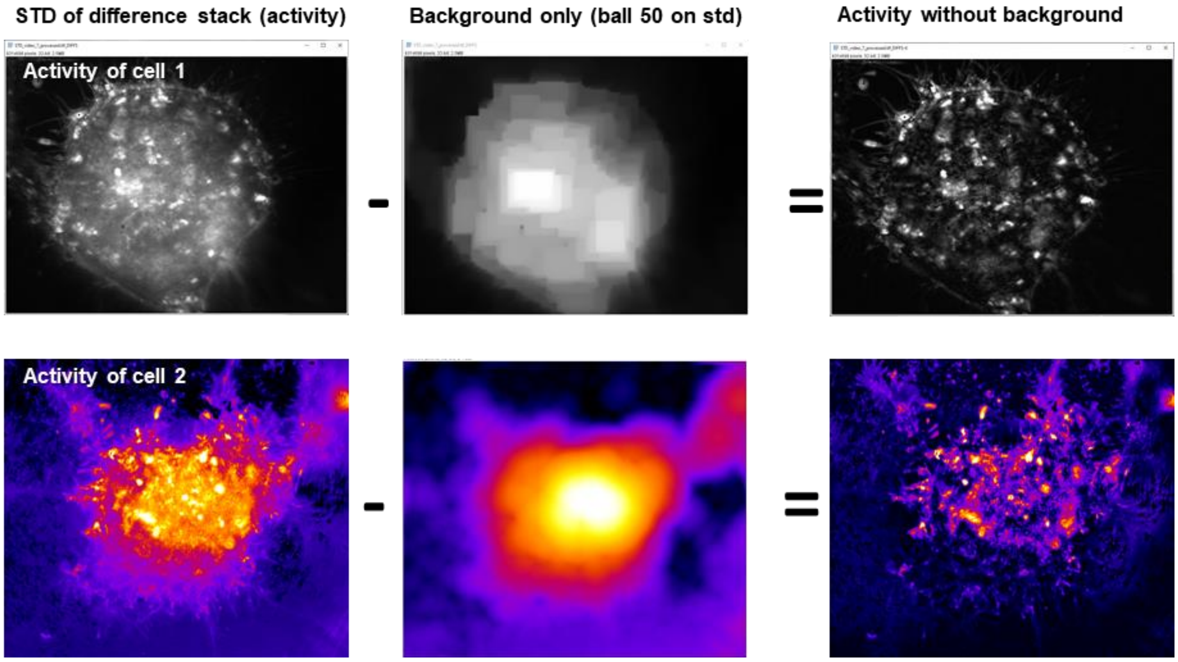

Figure S 8 | Effect of background subtraction on ROCS activities. - Supporting images to Figure 6. The activity of two H1299 lung epithelial cells (1 & 2) is analyzed by the standard deviation of 1000 difference images acquired within 10s. The activity of small LecA patches on the membrane or of endocytosed particles below the membrane can be better revealed by subtracting a smooth background of the standard deviation can be subtracted (rolling ball with radius 50 pixel).

A comparison of the TIR-F and TIR-ROCS images of a 90s-long continuous movie acquisition of two living J774 macrophages demonstrates the consistently good contrast in ROCS, which does not exhibit any decrease even after 90s of continuous illumination with blue light (see images and line profiles in Figure S 9). TIR-ROCS images are taken at 100 Hz with an exposure time of 10 ms resulting in 9000 images in total. Maximum gray values of 1600, together with the good signal-to-noise ratio, indicate that the exposure time or the laser power can still be significantly reduced (here: 1 mW). Short exposure times enable faster acquisition rates and less motion blur, reduced laser power reduces photodamage by reduced light dose. To assess potential phototoxicity, the cell activity was calculated from the TIR-ROCS time series (see main text for details). Here, time windows of 0.5s each were analyzed at the start of the recording and after 90s. We observe a mere 5% decrease in cell activity after 90s of constant illumination, thus concluding that the typical light dose we used in most experiments did not lead to significant phototoxic effects in J774 macrophages.

Supplementary Note 7: On photobleaching, phototoxicity and SNR in TIR-ROCS and TIR-F

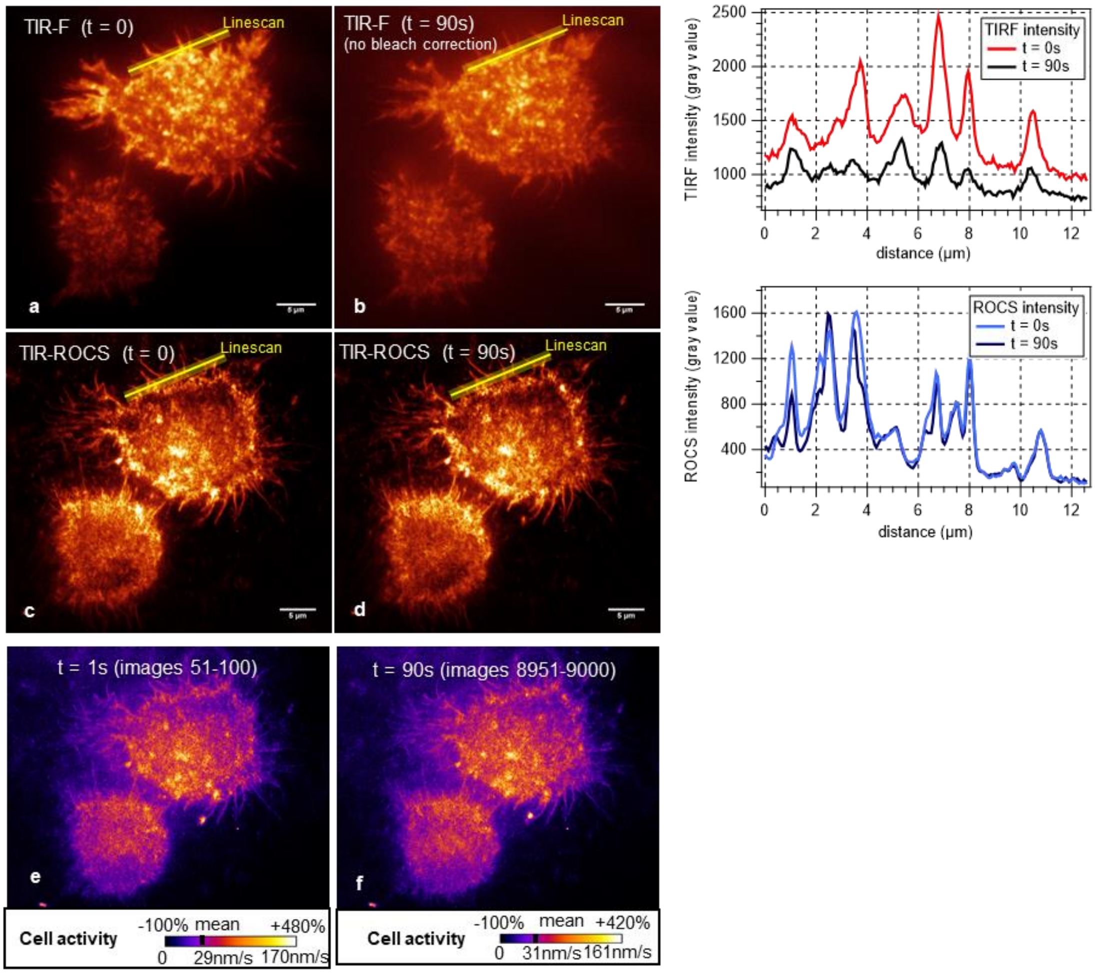

Figure S 9 | Continuous light exposure. TIR-F images of two living J774 macrophages, imaged in TIR-F mode at t = 0s (**a**) and after 90s (**b**). Same sample with TIR-ROCS imaging, 405nm laser source with maximum polar angle at t = 0s (**c**) and after 90s (**d**). All images are snapshots of continuous image sequences. **e,f** Cell activity maps of the TIR-ROCS time series, calculated within 0.5s time windows. The line profiles (yellow lines in the cell images) on the right side show the intensities in TIR-F and in TIR-ROCS images, respectively, at both time points.

Control images in TIR-F mode (lifeAct mCherry labelling) were taken with 561nm illumination wavelength, 200ms exposure time and P = 1mW). Comparing the line profiles in TIR-F and TIR-ROCS, it becomes apparent that TIRF requires a 20x longer exposure time (200 ms) to achieve similar gray levels as ROCS with an exposure time of 10ms. Furthermore, a significant amount of photobleaching is obvious (black linescan vs. red linescan), which limits the SNR of the TIRF movie significantly, which is no factor in ROCS.

Supplementary Note 8: On fluorescent protein expression and signal levels

Another drawback inherent to fluorescence imaging is the need for cumbersome sample modification with non-perfect transfection efficiency. As a consequence, some cells are well visible in fluorescence mode, whereas others with lower transfection efficiency remain dim or even dark (see Figure S 10 for demonstration). Although this effect can be minimized, depending on the type of fluorophore, transfection method and general experience, it can never be completely eliminated, making reliable fluorescent sample preparation in some cases a challenging task.

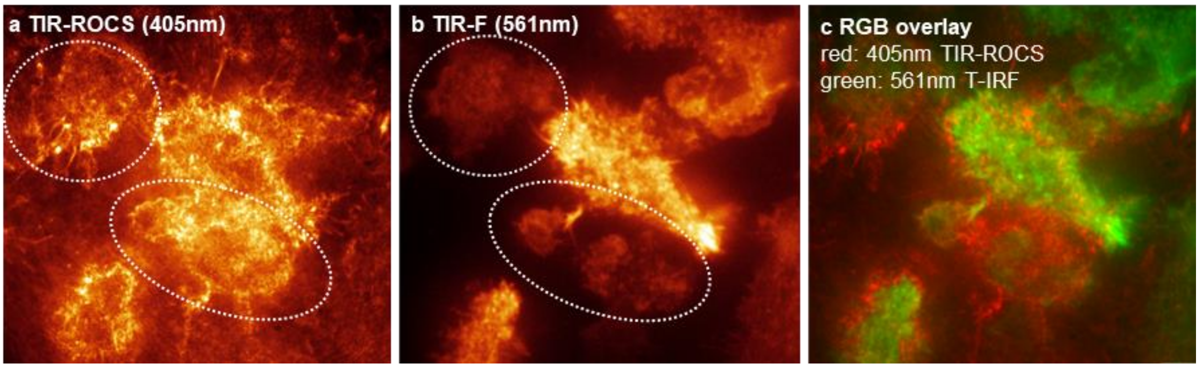

Figure S 10 | TIR-ROCS image of a dense J774 cell sample. **b** TIR-F image of the same sample (lifeAct-mCherry, 561nm excitation). **c** Red - green overlay of both images.
