# Supplementary material for: 100 Hz ROCS microscopy correlated with fluorescence reveals cellular dynamics on different spatiotemporal scales
Source: Nat Commun. 2022 Apr 1;13:1758. doi: 10.1038/s41467-022-29091-0 (PMC8975811; doi:10.1038/s41467-022-29091-0)
Supplement: Supplementary file 2 — Reporting Summary [file 41467_2022_29091_MOESM2_ESM.pdf]

nature research

Corresponding author(s): Alexander Rohrbach

Last updated by author(s): Feb 16, 2022

Reporting Summary

Nature Research wishes to improve the reproducibility of the work that we publish. This form provides structure for consistency and transparency in reporting. For further information on Nature Research policies, see our Editorial Policies and the Editorial Policy Checklist.

Statistics

For all statistical analyses, confirm that the following items are present in the figure legend, table legend, main text, or Methods section.

n/a	Confirmed	
☐	☒	The exact sample size (*n*) for each experimental group/condition, given as a discrete number and unit of measurement
☐	☒	A statement on whether measurements were taken from distinct samples or whether the same sample was measured repeatedly
☒	☐	The statistical test(s) used AND whether they are one- or two-sided<br>*Only common tests should be described solely by name; describe more complex techniques in the Methods section.*
☒	☐	A description of all covariates tested
☒	☐	A description of any assumptions or corrections, such as tests of normality and adjustment for multiple comparisons
☐	☒	A full description of the statistical parameters including central tendency (e.g. means) or other basic estimates (e.g. regression coefficient) AND variation (e.g. standard deviation) or associated estimates of uncertainty (e.g. confidence intervals)
☒	☐	For null hypothesis testing, the test statistic (e.g. *F*, *t*, *r*) with confidence intervals, effect sizes, degrees of freedom and *P* value noted<br>*Give P values as exact values whenever suitable.*
☒	☐	For Bayesian analysis, information on the choice of priors and Markov chain Monte Carlo settings
☒	☐	For hierarchical and complex designs, identification of the appropriate level for tests and full reporting of outcomes
☒	☐	Estimates of effect sizes (e.g. Cohen's *d*, Pearson's *r*), indicating how they were calculated

Our web collection on statistics for biologists contains articles on many of the points above.

Software and code

Policy information about availability of computer code

Data collection	ROCS images were recorded & transfered from mj2- to tiff- format by a routines written in MATLAB R2020b (Mathworks). Fluorescence images were recorder by software written in Python (Eclipse) 2.7.2 incl. NumPy and SciPy Packages.
Data analysis	Image data was analysed by Fiji (ImageJ) v1.53c, inkl. Plugins: TrackMate v. 7.5.1 and ImageJ mj2 file loader v1.0, Igor v8(Wavemetrics), MATLAB incl. Simulink and Image Acquisition Toolbox, Release R2020b , and TrackMate (ImageJ).

For manuscripts utilizing custom algorithms or software that are central to the research but not yet described in published literature, software must be made available to editors and reviewers. We strongly encourage code deposition in a community repository (e.g. GitHub). See the Nature Research guidelines for submitting code & software for further information.

Data

Policy information about availability of data

All manuscripts must include a data availability statement. This statement should provide the following information, where applicable:

- Accession codes, unique identifiers, or web links for publicly available datasets
- A list of figures that have associated raw data
- A description of any restrictions on data availability

Data supporting the main figures in this work are available at https://doi.org/…. Other data that support the findings of this study are available from the corresponding authors upon reasonable request. Further information regarding the experimental design may be found in the Nature Research Reporting Summary.

Field-specific reporting

Please select the one below that is the best fit for your research. If you are not sure, read the appropriate sections before making your selection.

☒ Life sciences ☐ Behavioural & social sciences ☐ Ecological, evolutionary & environmental sciences

For a reference copy of the document with all sections, see nature.com/documents/nr-reporting-summary-flat.pdf

Life sciences study design

All studies must disclose on these points even when the disclosure is negative.

Sample size	Sample sizes are determined by the number of experiments from which the standard deviations of the relevant measured quantities were no longer decreased.
Data exclusions	No data was excluded.
Replication	All attempts at replication were successful. Imaging experiments were repeated (replicated and performed independently) 5-10 times.
Randomization	Samples were allocated randomly.
Blinding	Investigators were blinded to group allocation during data collection and/or analysis.

Reporting for specific materials, systems and methods

We require information from authors about some types of materials, experimental systems and methods used in many studies. Here, indicate whether each material, system or method listed is relevant to your study. If you are not sure if a list item applies to your research, read the appropriate section before selecting a response.

Materials & experimental systems

n/a	Involved in the study	
☐	☒	Antibodies
☐	☒	Eukaryotic cell lines
☒	☐	Palaeontology and archaeology
☐	☒	Animals and other organisms
☐	☒	Human research participants
☒	☐	Clinical data
☒	☐	Dual use research of concern

Methods

n/a	Involved in the study	
☒	☐	ChIP-seq
☒	☐	Flow cytometry
☒	☐	MRI-based neuroimaging

Antibodies

Antibodies used	Monoclonal Anti-dinitrophenyl antibody (Clone SPE-7) (Sigma-Aldrich, D8406)
Validation	SPE-7 was used to decorate mast cells to induce IgE-mediated signalling and mast cell degranulation with DNP-HSA. Mast cell degranulation at the given concentration was validated by FACS measurements for avidin and CD63. Further references on manufacturer's website: https://www.sigmaaldrich.com/DE/de/product/sigma/d8406

Eukaryotic cell lines

Policy information about cell lines

Cell line source(s)	H1299 lung epithelial cell line, ATCC® CRL-5803, https://www.atcc.org/cell-products<br>J774A.1 Mouse macrophages (ATCC TIB-67, www.atcc.org/cell-products) transfected with pLife Act-TagGFP2 (ibidi GmbH, Germany)
Authentication	None of the cell lines were authenticated
Mycoplasma contamination	All cell lines were tested negative for mycoplasma.
Commonly misidentified lines (See ICLAC register)	No, commonly misidentified cell lines were not used.

Animals and other organisms

Policy information about studies involving animals; ARRIVE guidelines recommended for reporting animal research

Laboratory animals	6 to 10 week old male C57BL/6J mice (JAX 00664, The Jackson Laboratory) were used for the removal of bones and isolation of bone marrow (for primary mouse neutrophila and the culture of primary peritoneal mast cells).<br>New Zealand White rabbits (age ~1 year, mixed sex) were used for primary cardiomyocyte isolation.
Wild animals	No wild animals were used in this study
Field-collected samples	No field collected samples were used in the study
Ethics oversight	Animal breeding at the Max Planck Institute of Immunobiology and Epigenetics, as well as animal handling as the Institute for Experimental Cardiovascular Medicine, were conducted in accord with approval by the Regierungspräsdium Freiburg, Germany.<br>All experiments were carried out according to the guidelines stated in Directive 2010/63/EU of the European Parliament on the protection of animals used for scientific purposes, and approved by the local authorities in Baden-Württemberg (X-16/10R). New Zealand white rabbits were used in this study.

Note that full information on the approval of the study protocol must also be provided in the manuscript.

Human research participants

Policy information about studies involving human research participants

Population characteristics	Patient in sinus rhythm, male, age 73 years.
Recruitment	Tissue samples were obtained from tpatients undergoing open-heart surgery at the University Heart Center Freiburg - Bad Human atrial tissue sample was obtained from a 73-year old patient in sinus rhythm, undergoing open heart surgery at the University Heart Center Freiburg-Bad Krozingen. Informed consent was obtained prior to surgery. The patient was not compensated for their participation in the study.
Ethics oversight	The sample were processed via the CardioVascular BioBank (CVBB) Freiburg im Breisgau, as approved by the Ethics Committee of the University of Freiburg, Freiburg im Breisgau, Germany (CVBB ethical approval reference 393/16; study approval reference 60719).

Note that full information on the approval of the study protocol must also be provided in the manuscript.
