# Supplementary material for: 100 Hz ROCS microscopy correlated with fluorescence reveals cellular dynamics on different spatiotemporal scales
Source: Nat Commun. 2022 Apr 1;13:1758. doi: 10.1038/s41467-022-29091-0 (PMC8975811; doi:10.1038/s41467-022-29091-0)
Supplement: Supplementary file 4 — Description of Additional Supplementary Files [file 41467_2022_29091_MOESM4_ESM.pdf]

Title: Supplementary movie 1:
Description: Composition of an image of a J774 cell from 72 illumination directions within 10 ms. Left: individual coherent images. Right: Incoherent superposition of 72 images.

Title: Supplementary movie 2:
Description: 100 Hz ROCS movie of 2 myolid cells with high dynamics of vesicles.

Title: Supplementary movie 3:
Description: 100 Hz ROCS movie of 7 myolid cells with high dynamics of vesicles.

Title: Supplementary movie 4:
Description: Comparison of 10 Hz and 100 Hz ROCS movie of J j774 cells with many adherent filopodia. Right: Intensity difference between 10 Hz and 100 Hz acquisition rate.

Title: Supplementary movie 5:
Description: 100 Hz ROCS movie of two MK6 cells communicating with each other

Title: Supplementary movie 6:
Description: 100 Hz ROCS movie of ROI2 showing inter-cell communication through reorganization of protrusions

Title: Supplementary movie 7:
Description: 100 Hz ROCS movie of ROI3 showing fast reorganization of actin cortex of MK6 cell

Title: Supplementary movie 8:
Description: 100 Hz ROCS movie of two J774 cells with static adherent filopodia and highly dynamic dorsal filopodia.

Title: Supplementary movie 9:
Description: 100 Hz ROCS movie of ROI1 showing highly dynamic dorsal filopodia of J774 cells.

Title: Supplementary movie 10:
Description: 100 Hz ROCS movie of ROI2 showing single filopodium elongation through tip searching.

Title: Supplementary movie 11:
Description: 100 Hz ROCS composite movie of filopodia fluctuation and elongation. Right: Edges of filopodia

Title: Supplementary movie 12:
Description: 100 Hz ROCS movie showing retraction of filopodia backbones in J774 cells 8 sec after Latrunculin A injection.

Title: Supplementary movie 13:
Description: 100 Hz ROCS movie after ‘Find Edges’ operation (in ImageJ) showing retraction of filopodia backbones in J774 cell 8 sec after Latrunculin A injection.

Title: Supplementary movie 14:
Description: 100 Hz movie of subsequent ROCS image differences showing effect of inhibiting F-actin polymerization in J774 cell 7-9 sec after Latrunculin A injection.

Title: Supplementary movie 15:
Description: 100 Hz ROCS movie showing effect of inhibiting F-actin polymerization in J774 cell 6-13 sec after Latrunculin A injection.

Title: Supplementary movie 15:
Description: 100 Hz ROCS movie showing effect of inhibiting F-actin polymerization in J774 cell 6-13 sec after Latrunculin A injection.

Title: Supplementary movie 16:
Description: Two 100 Hz ROCS movies comparing the effect of the addition of DMSO solution without and with Latrunculin A.

Title: Supplementary movie 17:
Description: 100 Hz ROCS movie combined with 5 Hz fluorescence movie showing the effect of pore opening in IgE coated mast cells 10 sec after stimulating with DNP-HSA.

Title: Supplementary movie 18:
Description: 100 Hz movie of subsequent ROCS image differences as minimum projections (ImageJ) added up. Shown is the pore formation in a mast cell within only 1 sec.

Title: Supplementary movie 19:
Description: 100 Hz ROCS movie combined with 5 Hz fluorescence movie showing the granule release in direction parallel to the coverslip.

Title: Supplementary movie 20:
Description: 100 Hz ROCS movie of cardio fibroblasts with many fast fluctuating tunneling nanotubes (TNTs). Right: Movie of subsequent ROCS image differences.

Title: Supplementary movie 21:
Description: Composite movie of TNT motion activities changing within 4 seconds at the beginning (top row) and 10 minutes later (bottom row). Slow motion activities (0-10Hz, left column) a much lower than fast motion activities (10-100Hz, right column), which is not visible in supplementary movie 20.

Title: Supplementary movie 22:
Description: 100 Hz ROCS movie of diffusing and bound virus-sized particles and of macrophage J774 cells.

Title: Supplementary movie 23:
Description: 100 Hz ROCS movie of ROI3 showing diffusing and bound virus-sized particles at J774 cells with multiple filopodia.

Title: Supplementary movie 24: 1
Description: 00 Hz ROCS movie of ROI3 enables automatic particle tracking of particles in front of cell (highly visible in background).

Title: Supplementary movie 25:
Description: 100 Hz ROCS movie of ROI3 combined with 2 Hz fluorescence movie of labeled particles (flashing) allowing to compare particle contrast in front of cells imaged with ROCS.

Title: Supplementary movie 26:
Description: 100 Hz ROCS movie of H1299 cell (in gray) overlaid with 2Hz fluorescent movie of LecA clusters labeled with Cy5 (in orange).

Title: Supplementary movie 27:
Description: 100 Hz ROCS movie in ROI2 of H1299 cell (in gray) overlaid with 2Hz fluorescent movie of labeled LecA clusters (in orange) showing transport and endocytosis events.

Title: Supplementary movie 28:
Description: 100 Hz ROCS movie of H1299 cell (logarithmic intensity in gray) overlaid with 2Hz fluorescent movie of labeled LecA clusters (in orange) showing actin dynamics, transport and endocytosis.

Title: Supplementary movie 29:
Description: 100 Hz ROCS movie of H1299 cell in ROI2 revealing high dynamics of actin and LecA

clusters.

Title: Supplementary movie 30:
Description: composted movie of difference images of 100 Hz ROCS (left) and 2 Hz fluorescence (Cy5) revealing dynamics of H1299 cell and lecA clusters.

Title: Supplementary movie 31:
Description: 100 Hz ROCS movie of H1299 cell (log scale) revealing dynamics of LecA clusters in the membrane (focused structures) and in actin cortex (defocused structures).
